# Analysis of virulence factors of *Helicobacter pylori *isolated from a Vietnamese population

**DOI:** 10.1186/1471-2180-9-175

**Published:** 2009-08-23

**Authors:** Tomohisa Uchida, Lam Tung Nguyen, Akiko Takayama, Tadayoshi Okimoto, Masaaki Kodama, Kazunari Murakami, Takeshi Matsuhisa, Tuan Dung Trinh, Long Ta, Dang Quy Dung Ho, Hoa Hai Hoang, Tetsuko Kishida, Toshio Fujioka, Masatsugu Moriyama, Yoshio Yamaoka

**Affiliations:** 1 Department of Molecular Pathology, Oita University Faculty of Medicine, Yufu, Japan; 2 Department of Forensic Medicine, Oita University Faculty of Medicine, Yufu, Japan; 3 Department of Gastroenterology, Oita University Faculty of Medicine, Yufu, Japan; 4 Department of Gastrointestinal Endoscopy, Tama-Nagayama Hospital, Nippon Medical School, Tokyo, Japan; 5 Department of Pathology, Hospital 108, Hanoi, Vietnam; 6 Department of Hepato-Gastroenterology, Hospital 108, Hanoi, Vietnam; 7 Department of Endoscopy, Cho Ray Hospital, Ho Chi Minh, Vietnam; 8 Training and Researches Department, Cho Ray Hospital, Ho Chi Minh, Vietnam; 9 Department of Medicine-Gastroenterology, Michael E. DeBakey Veterans Affairs Medical Center and Baylor College of Medicine, Houston, Texas, USA; 10 Department of Environmental and Preventive Medicine, Oita University Faculty of Medicine, Yufu, Japan

## Abstract

**Background:**

The incidence of gastric cancer differs among countries in Asia, and it has been suggested that virulence factors associated with *Helicobacter pylori *are partly responsible. The aim of this study was to investigate several genetic factors regarded as virulence or molecular epidemiologic markers in *H. pylori *isolates from Vietnamese subjects.

**Results:**

The *cagA*, *vacA *and *cag *right-end junction genotypes of 103 *H. pylori *strains from Vietnam (54 from Hanoi and 49 from Ho Chi Minh) were determined by PCR and sequencing. Three types of deletion in the region located upstream of the *cagA *Glu-Pro-Ile-Tyr-Ala (EPIYA) repeat region were identified: the 39-bp deletion type, the 18-bp deletion type, and the no-deletion type. The majority of strains studied (77%; 80/103) had the 18-bp deletion irrespective of geographical location in the country or clinical outcome. All of the 39-bp and 18-bp deletion-type strains possessed the East Asian type *cagA *repeat region. The type II *cag *right-end junction genotype was predominant (84%). The *vacA *m1 genotype was significantly more common in strains isolated in Hanoi, where the incidence of gastric cancer is higher, than in strains from Ho Chi Minh.

**Conclusion:**

Pre-EPIYA-region typing of the *cagA *gene could provide a new genetic marker of *H. pylori *genomic diversity. Our data support the hypothesis that *vacA *m1 is closely associated with gastric carcinogenesis.

## Background

*Helicobacter pylori *is recognized to play a causative role in the pathogenesis of various gastroduodenal diseases including gastritis, peptic ulcer, gastric cancer and mucosa-associated lymphoid tissue (MALT) lymphoma [1-6]. However, only a minority of *H. pylori-*infected patients will develop severe manifestations, indicating that the clinical outcome is dependent on interactions between bacterial virulence, and host-related and environmental factors.

Gastric cancer is still a significant health problem in Asian countries. More than 56% of newly diagnosed gastric cancers arise in Asia, of which 42% are reported from China and 12% from Japan (data available at http://www-dep.iarc.fr/). However the incidence of gastric cancer varies greatly, even among different regions of Asia. Based on the age-standardized incidence rate (ASR) of gastric cancer, Asian countries can be categorized as high-risk (e.g., Japan, Korea, China), intermediate-risk (e.g., Vietnam) or low-risk (e.g., Thailand and Indonesia). In contrast, the prevalence of *H. pylori *infection is similar among these countries, being relatively high in the elderly population [7,8]. Thus, although the association between *H. pylori *infection and the development of gastric cancer has been well established, it is still unclear why there is such a wide variation in the incidence of gastric cancer among Asian countries, an issue that has been referred to as the "Asian enigma" or "Asian paradox" [7,9].

Recent molecular epidemiologic data suggest that genetic diversity of *H. pylori *might be partly responsible for this phenomenon. A large number of studies have investigated the roles of putative virulence factors of *H. pylori*, the best studied being the *cagA *and *vacA *genes. The structure of the 3' repeat region of the *cagA *gene varies between strains from Western countries and those from East Asian countries [10-17]; East Asian type *cagA *strains are reported to be more virulent than their Western counterparts [14,15].

*H. pylori *can be divided into five subtypes based on the structure of the right-end junction motif of the *cag *pathogenicity island (PAI), which can be a useful molecular marker for distinguishing isolates from different geographical areas [18]. Generally, type I is common in isolates from Western countries, type II in East Asian countries, and type III mainly in South Asia [18]. Types IV and V are relatively rare compared with the other types, but type V has been found in a few strains from India and Thailand [12].

There is considerable variation in vacuolation activity among *H. pylori *strains [19,20], primarily due to differences of *vacA *gene structure in the signal region (s1 and s2) and the middle region (m1 and m2) [21]. Among the s1 genotype, s1/m1 is toxic for a wider range of epithelial cells than s1/m2 [22]. The *vacA *s2/m2 strains are virtually non-toxic [21] and are rarely associated with diseases [23-25]. Importantly, most of the *H. pylori *strains isolated from countries with a high incidence of gastric cancer such as Japan and South Korea concurrently possess virulent genotypes such as *vacA *s1/m1 and East Asian type *cagA *[13,14]. In contrast, in countries with a low incidence of gastric cancer such as Thailand and India, a considerable proportion of *H. pylori *isolates have less virulent genotypes, such as *vacA *m2 and Western type *cagA *[12,13].

Vietnam is located on the borderline between regions with high and low risk of gastric cancer. Interestingly, the ASR of gastric cancer in Vietnam was 21.8 in 2002, which is considered to be intermediate (i.e., lower than Japan [62.0], Korea [69.7] and China [41.4], but higher than Thailand [4.3] and Indonesia [3.5]) http://www-dep.iarc.fr/. Moreover, despite the similarity in ethnicity and dietary factors, as well as the prevalence of *H. pylori *infection, the ASR of gastric cancer in the northern city of Hanoi is approximately 1.5 times higher than that in the southern city of Ho Chi Minh http://www-dep.iarc.fr/. We hypothesized that the *H. pylori *genotypes would differ between strains isolated from the two cities. Currently, however, there are few data about *H. pylori *genotypes isolated from Vietnam [26]. We therefore attempted to investigate several *H. pylori *genetic factors regarded as virulence or molecular epidemiologic markers in *H. pylori *isolates from Vietnam.

## Results

### Patients and *H. pylori*

We recruited a total of 103 Vietnamese patients (47 males and 56 females), aged 14 to 83 years (mean age, 45 years), of whom 54 were from Hanoi and 49 were from Ho Chi Minh. Twenty-five patients were judged to have peptic ulcer disease (16 from Hanoi and 9 from Ho Chi Minh) and 78 had chronic gastritis (38 from Hanoi and 40 from Ho Chi Minh).

### Classification of the *cagA *gene according to the pre-EPIYA region

We analyzed the sequences of the *cagA *Glu-Pro-Ile-Tyr-Ala (EPIYA) repeat region and upstream sequence of the EPIYA region of *H. pylori *isolated from Ho Chi Minh and Hanoi, located in the southern and northern parts of Vietnam, respectively. Except for five cases associated with *cagA*-negative strains, the EPIYA repeat region and pre-EPIYA region of the remaining 98 strains were successfully sequenced. The majority of Vietnamese strains (93%; 94/103) had an East Asian type EPIYA repeat with three EPIYA motifs (i.e., ABD type based on the previous classification [15,27]), and only 4 strains (4%) had a Western type EPIYA repeat with three EPIYA motifs (i.e., ABC type) (Table 1).

**Table 1:** Genotypes of *cagA *pre-EPIYA,*cagA *repeat, *cag *right-end junction and *vacA *of Vietnamese *H. pylori *strains.

		Total (n = 103)	Ho Chi Minh (n = 49)	Hanoi (n = 54)
*cagA *pre-EPIYA	Vietnamese pre-EPIYA type	80 (77%)	39 (80%)	41 (76%)
	East Asian pre-EPIYA type	13 (13%)	4 (8%)	9 (17%)
	Western pre-EPIYA type	5 (5%)	3 (6%)	2 (4%)
*cagA *repeat	East Asian type (ABD type)	94 (93%)	43 (88%)	51 (94%)
	Western type (ABC type)	4 (4%)	3 (6%)	1 (2%)
*cagA *(-)		5 (5%)	3 (6%)	2 (4%)
*cag *right-end	I	9 (9%)	8 (16%)	1 (2%)
	II	87 (84%)	37 (76%)	50 (93%)
	III	4 (4%)	2 (4%)	2 (4%)
	N.D.	3 (3%)	2 (4%)	1 (2%)
*vacA *s*	s1	103 (100%)	49 (100%)	54 (100%)
	s2	1 (1%)	0 (0%)	1 (2%)
*vacA *m†	m1	44 (43%)	15 (31%)	29 (54%)
	m2	54 (52%)	32 (65%)	22 (41%)
	N.D.	5 (5%)	2 (4%)	3 (6%)

N.D.: could not be determined.

* Both s1 and s2 were detected in one case.

† The prevalence of *vacA *m1 is significantly higher in Hanoi than in Ho Chi Minh, *p *< 0.05

**Figure 1:**
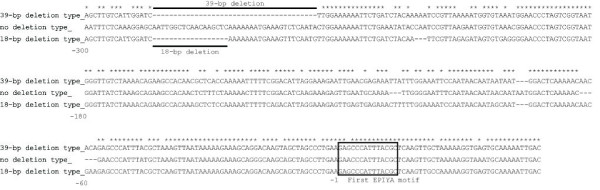
**Alignment of *cagA *pre-EPIYA region sequences from Vietnamese *H. pylori***. An 18-bp deletion, a 39-bp deletion, and no deletion were found at about 300 bp upstream of the first EPIYA region. The first EPIYA sequence is indicated in the clear square. Numbers were input from the first EPIYA motif.

Interestingly, about 300 bp upstream of the first EPIYA motif, we found that several strains carried a 39-bp or 18-bp deletion (Figure 1). All strains with the 39-bp and 18-bp deletion had an East Asian type EPIYA repeat and 4 of 5 (80%) strains without the deletion had a Western type EPIYA repeat. Thus, the East Asian type was subdivisible into two groups according to the upstream sequence of the EPIYA repeat region: the 39-bp and 18-bp deletion types. Importantly, the majority of Vietnamese strains (77%; 80/103) had the 18-bp deletion, irrespective of geographical location (80% in Ho Chi Minh and 76% in Hanoi) (Table 1). In contrast, only 13% (13/103) of the isolates carried the 39-bp deletion. In this study, we designated the 18-bp deletion type as the Vietnamese pre-EPIYA type, and the 39-bp deletion type as the East Asian pre-EPIYA type. Three types of pre-EPIYA region were distinguishable by simple PCR (data not shown) using primer sets covering the *cagA *pre-EPIYA region, as described in Methods. However, there was no relationship between pre-EPIYA types and clinical outcome in this Vietnamese population (data not shown).

### Genotypes of the *cag *right-end junction

It has been reported that the *cag *right-end junction motif can be classified into five groups [18]. We found that type II was the most common (84%), followed by type I (9%) and type III (4%) (Table 1). The remaining three strains could not be categorized into any genotype. This result was consistent with previous data showing that type II was the most common among *H. pylori *isolates from East Asian countries [13,18]. Interestingly, type I, which was considered to be specific for Western strains, was significantly more common in strains isolated in Ho Chi Minh (16%) than in those originating from Hanoi (2%) (p < 0.05). In contrast, type II was significantly more common in Hanoi (93%) than in Ho Chi Minh (76%) (p < 0.05). There was no significant relationship between the *cag *right-end junction types and clinical outcome in this Vietnamese population (data not shown).

Type II was very common in *H. pylori *strains carried by Vietnamese (86%: 69/80) and also in the East Asian pre-EPIYA type (100%: 13/13) (Table 2). In contrast, among strains with a Western pre-EPIYA type, type II accounted for 40% (2/5) and type I for the remaining 60% (3/5).

**Table 2:** Relationship between *cagA *pre-EPIYA type and *cag *right-end junction types or *vacA *genotypes.

		*cag *right-end junction type				*vacA *m type		
			
		I	II	III	N.D.	m1	m2	(-)
*cagA *pre-EPIYA type	Vietnamese (n = 80)	6	69	4	1	35	40	5
	East Asian (n = 13)	0	13	0	0	6	7	0
	Western (n = 5)	3	2	0	0	1	4	0
	*cagA *(-) (n = 5)	0	3	0	2	2	3	0

N.D.: not determined

### Genotypes of the *vacA *genotypes

All Vietnamese strains possessed the *vacA *s1 genotype and only one case from Hanoi possessed both the s1 and s2 genotypes, suggesting mixed infection with two strains. The m1 genotype was significantly more common in strains isolated in Hanoi than in those originating from Ho Chi Minh (54% vs. 31%) (p < 0.05) (Table 1).

The prevalence of the *vacA *m1 genotype was significantly higher in strains isolated from peptic ulcer patients (60%; 15/25) than in those from gastritis patients (37%; 29/78) (odds ratios: 2.59, 95% confidence interval: 1.00-6.68, p < 0.05). No significant relationship was observed between m region genotypes and pre-EPIYA deletion types (Table 2).

### *H. pylori *genotypes and histology

We examined whether the *vacA *genotypes and the *cagA *pre-EPIYA types were related to histological score. The five *cagA*-negative cases were excluded from histological analysis. Univariate analysis showed that the antral mononuclear cell infiltration scores were significantly higher in tissue infected with Vietnamese or East Asian pre-EPIYA types than in those infected with the Western type (Table 3). The East Asian *cagA *repeat type was highly associated with severe mononuclear cell infiltration (*p *< 0.01) and the type III *cag *right-end junction was associated with mild neutrophil infiltration (*p *< 0.01) (Tables 3 and 4). In contrast, there was no relationship between *vacA *middle-region genotypes and histological score (data not shown). There was no significant relationship between *cagA *genotypes and scores for atrophy and intestinal metaplasia (data not shown).

**Table 3:** Histological scores of mononuclear cell infiltration in patients with chronic gastritis infected with *H. pylori *strains of different *cagA *genotypesin the antrum.

	Mononuclear cell infiltration							
	
	pre-EPIYA typing			EPIYA repeat typing		*cag *right-end junction typing		
		
Grade	Vietnamese	East Asian	Western	East Asian	Western	I	II	III
none	0	0	0	0	0	0	0	0
mild	24	5	5	31	4	5	28	2
moderate	52	8	0	61	0	4	55	2
severe	4	0	0	4	0	0	4	0

p-value	* *p*	** *p*		*** *p*		N.S.		

Mann-Whitney rank sum test. N.S.: not significant.

* *p *< 0.01; ** *p *< 0.05; vs. Western type

*** *p *< 0.01

**Table 4:** Histological scores of neutrophil infiltration in patients with chronic gastritis infected with *H. pylori *strains of different cagA genotypes in the antrum.

	Neutrophil infiltration							
	
	pre-EPIYA typing			EPIYA repeat typing		*cag *right-end junction typing		
		
Grade	Vietnamese	East Asian	Western	East Asian	Western	I	II	III
none	4	1	1	7	0	0	4	3
mild	49	9	4	59	4	6	56	1
moderate	26	3	0	29	0	3	26	0
severe	1	0	0	1	0	0	1	0

p-value	N.S.			N.S.		** *p*	* *p*	

Mann-Whitney rank sum test. N.S.: not significant.

*p < 0.01; **p < 0.05; vs. type III

Multiple linear regression analysis was performed to determine which factor(s) was related to severity of histology. In the antrum, the *cag *end junction type III was significantly associated with milder neutrophil infiltration (partial regression coefficient [PRC] ± SE = -1.13 ± 0.35 compared with type I, *p *< 0.001) and more severe intestinal metaplasia (0.61 ± 0.27, *p *< 0.05) (Table 5). The PRC of -1.13 for the *cag *end junction type III for neutrophil infiltration suggests that the neutrophil infiltration score associated with *cag *end junction type III strains would be expected to be 1.12 points lower than with type I strains. The Western pre-EPIYA type was significantly associated with milder antral neutrophil infiltration (PRC ± SE = -0.66 ± 0.29 compared with the East Asian type, *p *<0.01).

**Table 5:** Multiple linear regression analysis of the severity of histology in the antrum.

	Types	Control	Case	PRC ± SE	*p *value
Neutrophil infiltration	*cag *right-end junction	type I	type II	0.017 ± 0.25	<0.001
			type III	-1.13 ± 0.35	
	*cagA *pre-EPIYA	East Asian	Western	-0.35 ± 0.30	0.08
			Vietnamese	0.19 ± 0.16	
Mononuclear cell infiltration	*cagA *pre-EPIYA	East Asian	Western	-0.66 ± 0.29	0.008
			Vietnamese	0.13 ± 0.15	
	*vacA *m	m2	m1	-0.20 ± 0.11	0.07
Atrophy	none				
Intestinal metaplasia	*cag *right-end junction	type I	type II	0.02 ± 0.17	0.03
			type III	0.61 ± 0.27	

PRC: partial regression coefficient

In the corpus and upper corpus, there were no significant differences between H. pylori genotypes and histological features, using either univariate analysis or multiple linear regression analysis (data not shown).

## Discussion

In this study, we identified three types of deletion located upstream of the *cagA *3' EPIYA repeat region: a 39-bp deletion, an 18-bp deletion, and lack of deletion. As of March, 2009, the GenBank database contained 326 *cagA *sequences of *H. pylori *that covered the pre-EPIYA region. Alignment of these sequences revealed that several strains carried a 39-bp or 18-bp deletion. As expected, the 39-bp deletion was present in most strains isolated from East Asia, but was absent in most strains from Western countries (Table 6). Moreover, all 19 *cagA *sequences with a unique 18-bp deletion type were present in Asian strains (Table 6), suggesting that the deletion patterns might be applicable as markers of genomic diversity among Asian *H. pylori *isolates. Although the 18-bp deletion type appears to be specific to Asian strains, the precise distribution was unclear because of the small number of cases examined. Among four Vietnamese *cagA *sequences deposited in GenBank, three had the 18-bp deletion type and one had the 39-bp deletion type (Table 6), suggesting that the 18-bp deletion type might be common in Vietnamese strains. GenBank data showed that the 18-bp deletion type also seemed to be common in Hong Kong and Thailand, in addition to Vietnam. However, our preliminary data showed that the prevalence of strains with the 18-bp deletion type was less than 10% in both Hong Kong and Thailand (our unpublished data). These data suggest that the 18-bp deletion type could be applicable as a new marker for Vietnamese *H. pylori *strains.

**Table 6:** Pre-EPIYA region patterns deposited in GenBank.

County	Total	39-bp deletion	No deletion	18-bp deletion
Japan	181	145 (80%)	31 (17%)	5 (3%)
China	37	33 (89%)	1 (3%)	3 (8%)
Korea	8	5 (63%)	3 (38%)	0
Hong Kong	8	4 (50%)	0	4 (50%)
Taiwan	5	4 (80%)	0	1 (20%)
Thailand	5	0	2 (40%)	3 (60%)
Vietnam	4	1 (25%)	0	3 (75%)
Sweden	16	0	16 (100%)	0
Colombia	14	0	14 (100%)	0
USA	13	0	13 (100%)	0
Italy	11	0	11 (100%)	0
Iran	8	0	8 (100%)	0
India	6	0	6 (100%)	0
Kazakhstan	3	0	3 (100%)	0
Germany	3	0	3 (100%)	0
Chile	1	0	1 (100%)	0
Austria	1	0	1 (100%)	0
Australia	1	0	1 (100%)	0
non-East Asia	1	0	1 (100%)	0

Total	326	192 (59%)	115 (35%)	19 (6%)

Through an extensive search of the Genbank database in combination with our data, we showed that these types, which were designated as the Western, East Asian and Vietnamese pre-EPIYA types, appear to be specific for each corresponding geographic region, and thus could be applicable as a new genetic marker for the genomic diversity of *H. pylori*. Interestingly, there was a close relationship between the *cagA *repeat region genotypes and the pre-EPIYA type. The great majority of the East Asian *cagA *repeat region type contained either the East Asian or Vietnamese pre-EPIYA type, whereas almost all of the Western *cagA *repeat region type had the Western pre-EPIYA type. Vietnamese strains could not be distinguished from other East Asian strains on the basis of previous genotyping including the *cagA *repeat region genotypes. In contrast, the novel pre-EPIYA types were able to distinguish Vietnamese strains from other East Asian strains with high sensitivity and specificity (e.g., sensitivity of 81.6% and specificity of 96.9% when the 98 *cagA*-positive Vietnamese strains in this study were compared with 162 Japanese strains deposited in GenBank). Therefore, this novel system will be useful for epidemiological studies of the distribution of Vietnamese strains. Notably, the Vietnamese pre-EPIYA type is predominant in Vietnam, where the incidence of gastric cancer is lower than in other East Asian countries such as Japan and South Korea, suggesting that the pre-EPIYA region might have some biological functions that partly contribute to the differences in incidence of gastric cancer, although we were unable to find any differences in the prevalence of peptic ulcer disease and histological findings between East Asian and Vietnamese pre-EPIYA types in this study. Further studies will be necessary to investigate the function of the pre-EPIYA region.

On the basis of structure, the *cag *right-end junction is classifiable into five subtypes [18]. Generally, type I is common in isolates from Western countries, type II in East Asian countries, and type III mainly in South Asia [18]. In agreement with previous data [12,13,18], the majority of Vietnamese strains we studied were type II strains. Interestingly, 16% of strains isolated in Ho Chi Minh possessed type I, which was a much higher prevalence than in other East Asian strains (e.g., none of 449 strains from Japan, Korea, Taiwan or Hong Kong possessed type I in a previous study [13]). This might explain the relatively higher frequencies of East Asian-type *cagA *amongst Hanoi isolates (e.g. East Asian pre-EPIYA and *cagA *repeat types), and hence the higher incidence of gastric cancer in that population. However, the reason for the high prevalence of type I in Ho Chi Minh is currently unknown. There is no evidence for a greater level of interracial mixing with Europeans in Ho Chi Minh than in Hanoi, particularly during the latter half of the 20th century. Extensive surveys will be necessary to elucidate the geographical distribution in East Asian countries. Interestingly, histological data from the antrum showed that the *cag *end junction type III was significantly associated with mild neutrophil infiltration and severe intestinal metaplasia. This is the first study to have demonstrated a relationship between *cag *end junction type and histological features; however the number of type III strains in this study was very small (n = 4) and further work will be necessary to clarify the importance of type III genotypes in countries where the prevalence of type III is high (e.g., South Asia).

The multifactorial model of gastric malignant transformation is currently accepted, and not only *H. pylori *virulence factors, but also other factors such as host genetic susceptibility and environmental factors will undoubtedly play certain roles. In Vietnam, the incidence of gastric cancer in the northern city of Hanoi is reported to be 1.5 times higher than that in the southern city of Ho Chi Minh. Importantly, the two cities share a lot of similarity in terms of ethnicity, living standards, lifestyle and dietary habits. Therefore, these two cities can serve as a good model for understanding the role *H. pylori *virulence factors in the development of gastric cancer. In this study, the prevalence of the *vacA *m1 type, which is currently considered to be more toxic and more closely associated with the development of gastric cancer than the m2 type, was significantly higher in strains isolated in Hanoi than those originating from Ho Chi Minh. Interestingly, compared with other East Asian countries such as Japan and Korea, where the incidence of gastric cancer is high, the prevalence of the *vacA *m1 type in Vietnam is much lower [13]. Taken together, our data support the hypothesis that the *vacA *m1 genotype is closely associated with gastric carcinogenesis and may provide a partial explanation for the Asian paradox. In addition, we have also found that the *vacA *m1 genotype was related to the development of peptic ulcers in the Vietnamese population. Although we failed to obtain evidence that m1 strains induced more severe gastric injury in terms of histology, our current data support the hypothesis that m1 strains are more toxic than m2 strains, and that the m1 genotype play a major role in countries where other factors are relatively homogeneous. Overall, we propose that examination of *H. pylori *genotypes in strains isolated from two cities in Vietnam, Ho Chi Minh and Hanoi, would be useful for investigating the roles of *H. pylori*-related factors in the pathogenesis of gastroduodenal disease.

## Conclusion

We have found three types of deletion in the pre-EPIYA region of the *cagA *gene: a 39-bp deletion, an 18-bp deletion, and no deletion, and demonstrated that the *cagA *genotype could be applicable as a new genetic marker of genomic diversity in *H. pylori*. In fact, the 18-bp deletion type appeared to be a marker of Vietnamese *H. pylori*. Comparison of two geographically distant cities in Vietnam, Hanoi and Ho Chi Minh, showed that the *vacA *m1 genotype, thought to be more toxic than the *vacA *m2 type, is more prevalent in Hanoi, where the incidence of gastric cancer is higher than in Ho Chi Minh. Our data support the hypothesis that the *vacA *m1 type is closely associated with gastric carcinogenesis.

## Methods

### Patients and *H. pylori*

*H. pylori *strains were obtained from the gastric mucosa of *H. pylori*-infected patients who underwent endoscopy at 108 Hospital, Hanoi, and Cho Ray Hospital, Ho Chi Minh. The biopsy specimens were immediately placed in Portagerm pylori (BioMérieux, Nürtingen, Germany)[28] at 4°C and then sent to Oita University, Oita, Japan. *H. pylori *was cultured as described previously [14]. Informed consent was obtained from all participants and the protocol was approved by the local hospital ethics committees. Patients with a history of partial gastric resection, *H. pylori *eradication therapy or treatment with antibiotics, bismuth-containing compounds, H2-receptor blockers or proton pump inhibitors within 4 weeks prior to the study were excluded.

### *H. pylori *genotyping

For DNA extraction, multiple colonies on blood agar plates were harvested together, and bacterial genomic DNA was extracted according to the CTAB (hexadecyltrimethylammonium bromide) method [29] and subsequently suspended in TE buffer (10 mM Tris HCl and 1 mM EDTA). A DNA fragment covering approximately 300 bp upstream from the first EPIYA motif in the *cagA *3' repeat region, which we designated the pre-EPIYA region in this study, was amplified by PCR using the following primer sets: T5: 5'-AAG CGT TAG CCG ATC TCA AA-3' (forward), and 1-AS: 5'-CAT TAC CGA CTA GGG TTC C-3' (reverse) [27]. The amplified DNA fragments were separated by electrophoresis on 2% agarose gel, stained with ethidium bromide, and finally visualized under ultraviolet light.

For sequencing of the pre-repeat region of the *cagA *gene, a DNA fragment of approximately 1,100 bp covering both the pre- EPIYA region and repeat region was initially amplified by PCR using the following primer sets: 2059f: 5'-GAA TTG TCT GAT AAA CTT G-3' (forward), and 3156r: 5'-GCG TAT GTG GCT GTT AGT AGC G-3' (reverse), then the amplified DNA fragments were sequenced with an ABI Prism 310 Genetic Analyzer [27] (Applied Biosystems, CA) in accordance with the manufacturer's instructions. Multiple sequence alignments of the *cagA *pre-EPIYA sequences were generated using the ClustalX programs (downloaded from ftp://ftp.ebi.ac.uk/pub/software/clustalw2).

The *vacA *genotyping (signal regions s1 and s2, and middle regions m1 and m2) and *cag *right-junction motif genotyping (type I to V) were performed as described previously [11,18,21].

### Dot blot analysis

To confirm the *cagA *negative status, dot blot analysis was performed as described previously[30]. Briefly, 200 ng of each sample DNA was mixed with denaturing buffer and spotted onto a Hybond N^+ ^membrane (Amersham Biosciences, Buckinghamshire, UK) using a 96-well Bio-Dot apparatus (Bio-Rad, Ivry-sur-Seine, France). DNA of the reference strain ATCC43504 and human DNA were also transferred to the membrane as positive and negative controls, respectively. The *cagA *of strain ATCC43504 was amplified by PCR with the above-mentioned primer sets. The amplified fragments were purified with an Illustra GFX PCR DNA and Gel Band Purification Kit and used as probes. The probes were labeled with horseradish peroxidase, hybridized to the membranes overnight at 42°C, and finally exposed to Hyperfilm ECL using ECL Direct Nucleic Acid Labeling and Detection Systems (Amersham Biosciences, Buckinghamshire, UK).

### Histological analysis

Three biopsy specimens from the antrum, corpus and upper part of the lesser curvature were used for histological examination. The biopsy specimens were fixed in 10% buffered formalin, and thinly sliced sections were stained with hematoxylin and eosin (H&E) and Giemsa. Histological features of neutrophil infiltration, mononuclear cell infiltration, grade of atrophy and grade of intestinal metaplasia were scored into four grades in accordance with the Updated Sydney system (0: none, 1: mild, 2: moderate, 3: severe) [31].

### Statistical analysis

Statistical analysis of the distribution of *H. pylori *genotypes was performed using Fisher's exact test. The Mann-Whitney rank sum test was used for assessing differences between ordered categories such as histological grade. The effects of the *H. pylori *genotypes on the risk for developing peptic ulcer in patients were expressed as odds ratios with 95% confidence intervals with reference to subjects with gastritis. Multiple linear regression analysis was performed to determine which factor(s) was related to the severity of histology, where age, sex, bacterial factors and clinical outcome were explanatory variables. Variables were selected by backward stepwise deletion in the logistic regression and by the F-out and F-in stepwise method in the linear regression, where F values were both 2.0. Differences at *P *< 0.05 were accepted as statistically significant. Calculations were carried out using the statistical software package ''JMP IN(R) 5.1J'' (SAS Institute, Cary, NC) or ''HALBAU'' (Gendai Sugaku-sha, Kyoto, Japan).

Nucleotide sequence data reported are available under the DDBJ accession numbers AB469377, and AB469561 to AB469657.

## Authors' contributions

TU participated in the design of the study, carried out the experiments and drafted the manuscript. LTN and AT carried out the PCR experiments and statistical analysis. TM, TDT and LT arranged the patients and performed endoscopy in Hanoi. DQDH, HHH and TO arranged the patients and performed endoscopy in Ho Chi Minh. MK, KM and TK participated in the discussion of the study design. TF, MM and YY designed the study. All authors have read and approved the final manuscript.
